# Supplementary material for: Human Induced Pluripotent Stem Cell-Derived Cardiomyocyte Encapsulating Bioactive Hydrogels Improve Rat Heart Function Post Myocardial Infarction
Source: Stem Cell Reports. 2017 Oct 5;9(5):1415–22. doi: 10.1016/j.stemcr.2017.09.003 (PMC5830963; doi:10.1016/j.stemcr.2017.09.003)
Supplement: Document S1. Supplemental Experimental Procedures and Figures S1–S4 [file mmc1.pdf]

Stem Cell Reports, Volume *9*

Supplemental Information

Human Induced Pluripotent Stem Cell-Derived Cardiomyocyte Encapsulating Bioactive Hydrogels Improve Rat Heart Function Post Myocardial Infarction

Andre Chow, Daniel J. Stuckey, Emaddin Kidher, Mark Rocco, Richard J. Jabbour, Catherine A. Mansfield, Ara Darzi, Sian E. Harding, Molly M. Stevens, and Thanos Athanasiou

Figure S1

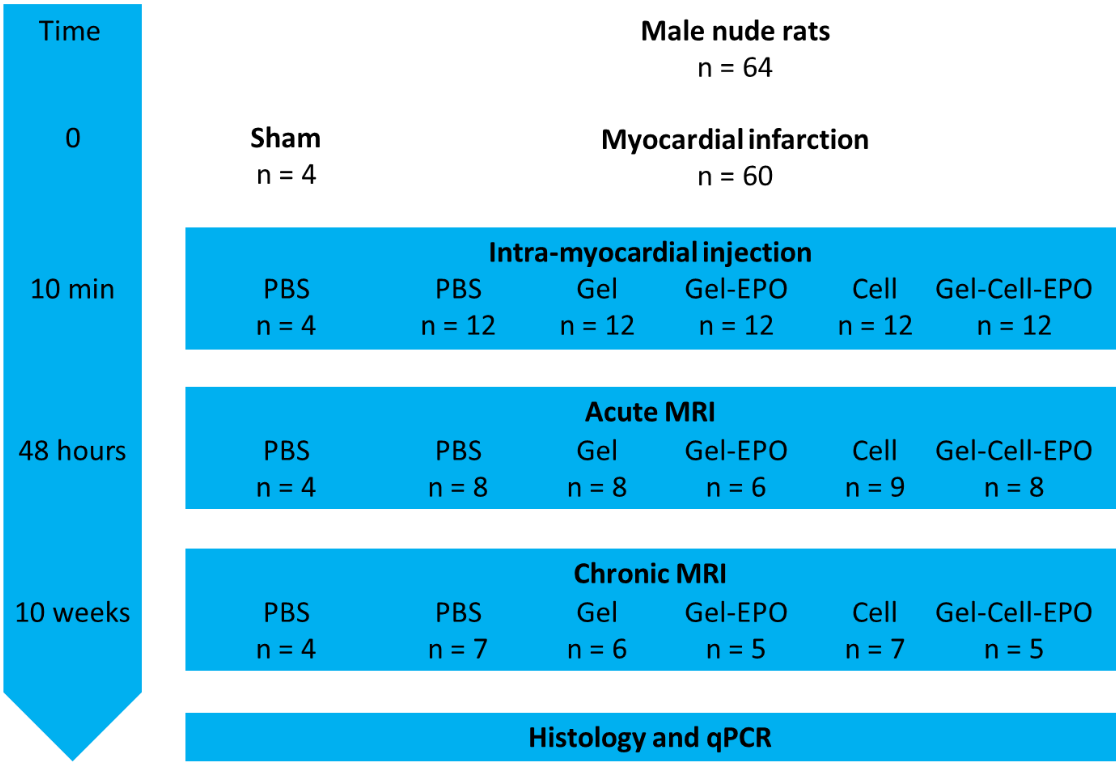
Time
0
10 min
48 hours
10 weeks
Male nude rats
n = 64
Sham
n = 4
Myocardial infarction
n = 60
Intra-myocardial injection
PBS
n = 4
PBS
n = 12
Gel
n = 12
Gel-EPO
n = 12
Cell
n = 12
Gel-Cell-EPO
n = 12
Acute MRI
PBS
n = 4
PBS
n = 8
Gel
n = 8
Gel-EPO
n = 6
Cell
n = 9
Gel-Cell-EPO
n = 8
Chronic MRI
PBS
n = 4
PBS
n = 7
Gel
n = 6
Gel-EPO
n = 5
Cell
n = 7
Gel-Cell-EPO
n = 5
Histology and qPCR

Figure S2

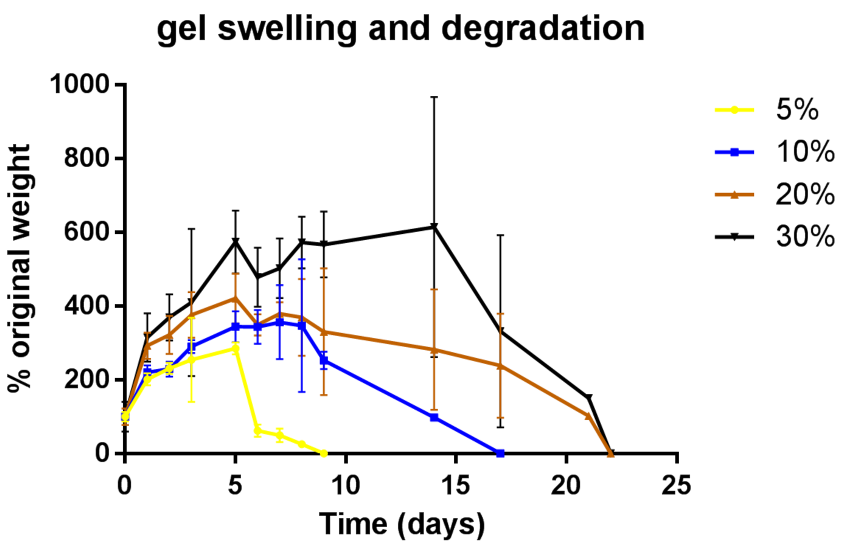

Figure S2. Hydrogel swelling and degradation

300 μl of PEG hydrogels (PEG4A + PEGDT) were formed at 5%, 10%, 20% and 30% (n = 4 individual replicates for each group), set overnight and immersed in 1ml of PBS. Wet weight of the gels was measured up to 22 days and expressed as a percentage of the original weight. Hydrogels of higher weight/volume concentrations swelled to a greater extent and took longer to degrade than lower concentration hydrogels. Data are presented as mean ± S.D.

Figure S3

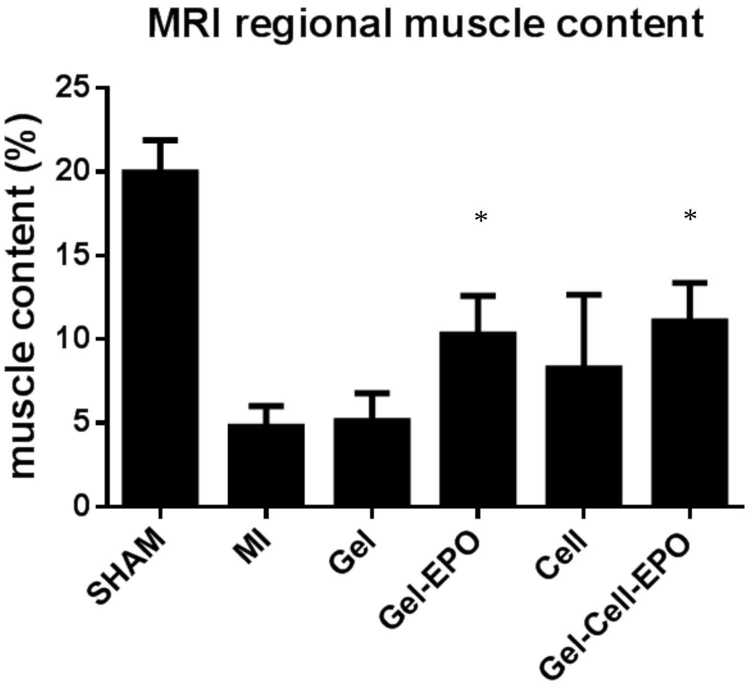

Figure S3. Regional muscle content within the infarct region

Myocardial viability within the apical (infarcted) region of the LV was measured as the non-enhanced area within apical LGE images and expressed as percentage viable tissue relative to the total left ventricular mass (to normalize for post infarct hypertrophy). All infarcted hearts had less viable tissue than sham animals. Hearts treated with GEL-EPO and GEL-CELL-EPO had significantly more viable tissue within the infarcted region than untreated infarcts. Data are presented as mean ± S.D. Statistically significant difference between group versus MI (* $p < 0.05$);

Figure S4

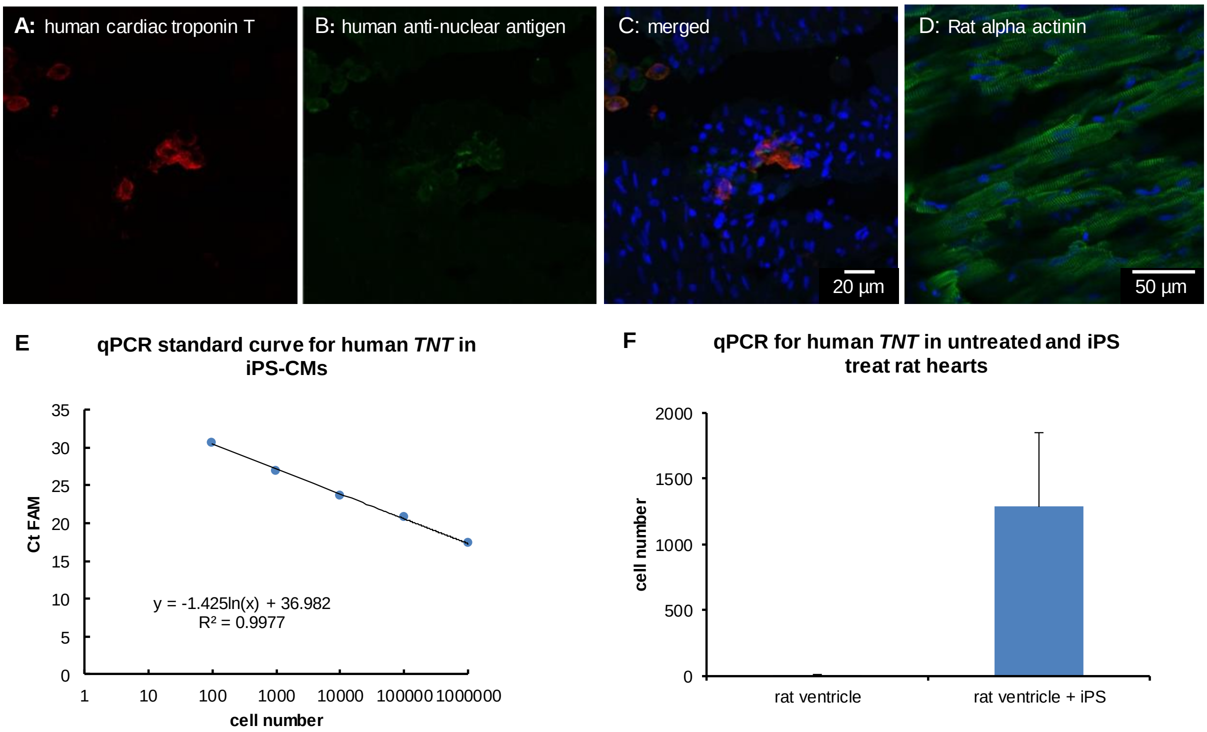

Figure S4. Immunohistochemical staining and qPCR of treated rat hearts

At 10 weeks after grafting immunohistochemical staining for human cardiac troponin T within iPS-CM grafted rat tissue was unable to identify any engrafted human cells. To confirm that human iPS-CMs could be detected within rat heart tissue using our protocol, we stained for human cardiac troponin T and human anti-nuclear antigen shortly after injection of iPS-CM into rat hearts. Human cardiac troponin T (A) and human anti-nuclear antigen (B) positive cells were readily identified within the tissue. Rat specific cardiomyocyte staining with alpha actinin is also presented (D). At 10 weeks after grafting qPCR for human cardiac markers within iPS-CM grafted rat tissue was unable to identify any engrafted human cells. To confirm that human iPS-CMs could be detected within rat heart tissue using our protocol, we generated a qPCR standard curve for human *TNT* from serial dilution on human iPS-CMs (E) and then used the same qPCR protocol to quantify cell content shortly after injection of 500 000 iPS-CM into rat hearts. Human *TNT* was detected in all hearts injected with iPS-CM (F), while untreated rat ventricular tissue had no human *TNT*. Data are presented as mean ± S.D.

Supplementary methods

Hydrogel preparation and materials

Four-arm polyethylene glycol (PEG) acrylate (PEG4A: Average MW = 23,300) and PEG dithiol (PEGDT: Average MW = 1,019) were purchased from Laysan Bio (Arab, AL, USA) and Sigma-Aldrich (Dorset, England) respectively. Basic PEG hydrogels were formed using a Michael addition reaction by adding PEGDT (dissolved in 1% triethanolamine) to PEG4A (dissolved in phosphate buffer saline).

Viscoelastic properties of the hydrogels were assessed using small amplitude oscillatory shear experiments on a rheometer (AR 2000ex, TA Instruments, Crawley, West Sussex, England) with parallel plate geometry (8 mm to 25 mm) at 37°C. Gel properties were examined by frequency sweep experiments at 1-10 hz (ω = 6.3–62.83 rad/s) at a fixed strain amplitude of 0.001 (0.1%) in order to ensure uniform mechanical properties were maintained at frequencies present in the human (1 hz, 6.3 rad/s) and rat (8 hz, 2.3 rad/s) heart.

Cell culture

Induced-pluripotent stem cell derived cardiomyocytes (iPS-CM) were purchased from Cellular Dynamics Inc (Madison, WI, USA) and have been extensively characterized by the supplier. From the 35 batches we received, the mean ± SD purity reported by suppliers was 98.2 ± 1.1% based on α-MHC staining, with no batches under 95%. Cells were thawed according to manufacturer's instructions and plated onto 0.1% (w/v) gelatin coated surfaces at a density of ~55,000 cells per cm^2. This allowed cultures to form a spontaneously beating syncytium of cardiomyocytes. Approximately 500,000 cells per rat were delivered by direct intramyocardial injections (see below). Cell viability after passing cells through the 27G needle used for injection was >90% as tested using trypan blue exclusion. Although this cell number is lower than that used in some other iPS-CM grafting studies, it is equivalent to many small animal and large animal experiments when scale is taken into consideration [1-4].

Animal model of myocardial infarction

All animal experiments were conducted under Home Office Project License 70/6568 and in accordance with local the Imperial College Ethics Committee and the ARRIVE Guidelines on animal research. Seven-week old male nude rats (approximately 150-200 g) were purchased from Charles River Limited (Germany), and allowed to acclimatize for 7 days prior to experiments. Prior to commencing surgery prophylactic antibiotics (Enrofloxacin 5 mg/kg intramuscular injection) were administered and analgesia (Buprenorphine 0.1 mg/kg intramuscular injection) was given. Induction of anaesthesia was performed by inhalation of a 5% Isoflurane in oxygen, then intubated and

ventilated using a Harvard Small Animal Ventilator (Model 683, Harvard Apparatus, Massachusetts, USA) at a ventilation rate of 80-90 breaths per minute, a tidal volume of 1.5-2.0 ml, and 2 - 2.5% isoflurane in oxygen. The thoracic cavity and heart were exposed via a left thoracotomy incision in the 3rd or 4th left intercostal space. A 7/0 prolene suture (Ethicon Inc., NJ, USA) on a round bodied curved needle was used to ligate the proximal part of the left anterior descending artery (LAD), approximately 1-2 mm distal to the inferior border of the left atrium, along an axis parallel to the atrioventricular groove. Successful ligation of the LAD was demonstrated by blanching and cyanosis of the myocardium distal to the tied suture, indicating myocardial ischemia. Sham surgery was performed by passing a suture and needle through the myocardium without ligation.

Injection of therapeutics

A 10% (w/v) 4-Armed PEG acrylate solution was prepared by 1:1 mixing of 20% 4-Armed PEG acrylate solution with cell suspension and the appropriate volume of 10% PEG dithiol was added to this mixture just prior to injection to initiate the gelling process. The final mixture was drawn up into a 1 ml syringe with 27G needle. Following successful infarction, hydrogels, cells, or a hydrogel and cell mixture was injected by 3 x 50 µl injections around the infarct border zone through a 27G needle. Maintenance anesthesia was reduced to 1.0% Isoflurane, and the chest wall was closed using a 4/0 Ethibond suture.

The experimental groups were as follows:

Sham: Sham operated control group that received PBS injection

MI: Myocardial infarction group that received PBS injection

Gel: MI group that received 10% PEG hydrogel injection

Gel-EPO: MI group that received 10% PEG hydrogel containing 1unit/ml EPO

Cell: MI group that received 5x10^5 iPS-CMs in PBS

Gel-Cell-EPO: MI group that received 10% PEG hydrogel containing 1unit/ml EPO and 5x10^5 iPS-CMs

Cardiac MRI

Anaesthetized rats were put under ECG with respiration monitoring and placed within a 4.7 Tesla DirectDrive Varian MRI System (Palo Alto, CA, USA). Cardiac and respiratory-gated cine-MRI was performed in the true short-axis orientation and covered the whole left ventricle (LV) (1.5 mm slice thickness, TE/TR 1.6/5 ms; 17.5° pulse; field of view 51.2 × 51.2 mm; matrix size 128 × 128; voxel size 400 × 400 × 1500 µm; 25 to 35 frames per cardiac cycle, 3 signal averages). Infarct size was

assessed by late gadolinuium enhanced (LGE) MRI performed 25 minutes after intraperitoneal injection of 0.5 mg/kg Gd-DTPA-BMA (Omniscan) using a multi slice inversion recovery sequence (1 mm slice thickness, TE/TR 1.6/3.2 ms; 90° excitation pulse; field of view 51.2 × 51.2 mm; matrix size 192 × 192; voxel size 266 × 266 × 1000 µm, 1 signal average) [5]. Data were analysed using Image J software (National Institutes of Health). End diastolic and end systolic volumes were measured by semi-automated segmentation of each slice and ejection fractions (EF) were calculated as (end diastolic volume - end systolic volume) / end diastolic volume. LV mass was measured by semi-automated segmentation of the myocardial area in all slices of the inversion recovery acquisition multiplied by the slice thickness (1 mm) and the specific gravity of the myocardium (1.05). Infarct mass was measured in every slice as the area of myocardial tissue with signal intensity >2 SD above signal intensity of remote tissue, multiplied by the slice thickness (1 mm) and the specific gravity of the myocardium (1.05). Infarct surface area was measured as the epicardial length of the enhanced tissue multiplied by the slice thickness, and was also expressed relative to the epicardial circumference of the whole LV (LGE surface area %). Myocardial viability within the apical (infarcted) region of the LV was measured as the non-enhanced area within apical LGE images and expressed as percentage viable tissue relative to the total left ventricular mass (to normalize for post infarct hypertrophy). MRI was performed within 48 hours of surgery and cell delivery, and repeated at 10 weeks. Primary outcome measures were LV end-diastolic and end-systolic chamber volumes, stroke volume and EF and LGE infarct size. MRI acquisition and analysis was performed by an investigator blinded to the experimental groups.

Histology

Ten weeks after infarction, animals were sacrificed; hearts were fixed by intraventricular infusion of 10% (v/v) formalin, explanted, and kept in 10% formalin for 24 hours followed by 30% (w/v) sucrose solution for at least 24 hours. Hearts were embedded in Optimal Cutting Temperature compound, frozen in liquid nitrogen-cooled isopentane baths and 10 µm sections were cut using a cryotome. For immunostaining studies, negative control samples were taken from the MI group, and positive control samples were from human tissue taken from patients with dilated cardiomyopathy, as well as iPS-CM grown on glass coverslips. Primary antibodies for human ANA (1 in 200, MAB1281, Millipore) and troponin (1in 250, AB45932, Abcam) were then incubated for 2 hours, washed in D-PBS and then incubated with secondary antibodies (Alexa fluor 488 and 546, 1 in 500) for 1 hour. After further washing, sections were incubated with Hoechst (1 in 1000) for 10 minutes.

Images of the infarct zone (x4 magnification) were analyzed using ImageJ software. This allowed measurement of infarct thickness, as well as measurement of the proportion of the infarct stained red by Massons Trichrome. This allowed calculation of the amount of muscle fibres present in the infarct zone. Fluorescence microscopy was used to image samples labeled with immunostaining.

Quantitative Polymerase Chain Reaction (qPCR)

The infarct zone and remaining left ventricular wall was excised from ~3 mm thick frozen heart sections and approximately 30 mg of each were placed separately into ice-cold TRI Reagent (Sigma-Aldrich). Tissue samples were disrupted and homogenized in the TRI Reagent solution using a rotor-stator homogenizer. Following homogenization, total RNA was extracted from cardiac tissue samples using a RNeasy Mini Kit (Qiagen) according to the manufacturer's specifications and cDNA was reverse transcribed from normalized amounts of RNA, as determined by absorbance at 260 nm (A_{260}) in a NanoDrop 8000 spectrophotometer. A similar protocol was used for the acute cell retention studies presented in Figure S4 except 50 – 100 mg of tissue was used. All RNA procedures were conducted in an RNase-free environment. All freshly synthesized cDNA was stored overnight at 4°C. The following day, real-time PCR was performed in technical triplicate using pre-validated TaqMan® assays from Life Technologies™ in a Rotor Gene 6000 (Corbett Life Science) according to the manufacturers' instructions.

Infarct zones and remaining left ventricular areas were analyzed separately. Negative control samples were taken from the sham and MI groups. Positive control samples used were human samples from patients with dilated cardiomyopathy. qPCR was performed for an assortment of early and late cardiac markers, including atrial natriuretic factor (*NPPA,* Hs00383230_g1*)*, myosin-6 (*MYH6,* HS01101425_M1), myosin-7 (*MYH7,* HS01110632_M1), alpha-1A and 1B adrenergic receptors (*ADRA1a,* HS00169124_M1 and *ADRA1b,* HS00171263_M1) and cardiac troponin T (*TNT,* HS00165960_M1).

Statistical Analysis

Results were tabulated using Microsoft Excel and Graphpad Prism. A one-way between groups ANOVA ($\alpha = 0.05$) was performed for group comparison. If the results of the ANOVA were found to be significant, post-hoc analysis was performed using the Tukey multiple comparisons test to compare results between all groups. Data are presented as mean ± SEM.

References

1. Miao Q, Shim W, Tee N, Lim SY, Chung YY, Ja KP, Ooi TH, Tan G, Kong G, Wei H *et al*: **iPSC-derived human mesenchymal stem cells improve myocardial strain of infarcted myocardium**. *Journal of cellular and molecular medicine* 2014, **18**(8):1644-1654.
2. Citro L, Naidu S, Hassan F, Kuppusamy ML, Kuppusamy P, Angelos MG, Khan M: **Comparison of human induced pluripotent stem-cell derived cardiomyocytes with human mesenchymal stem cells following acute myocardial infarction**. *PloS one* 2014, **9**(12):e116281.
3. Shiba Y, Gomibuchi T, Seto T, Wada Y, Ichimura H, Tanaka Y, Ogasawara T, Okada K, Shiba N, Sakamoto K *et al*: **Allogeneic transplantation of iPS cell-derived cardiomyocytes regenerates primate hearts**. *Nature* 2016, **538**(7625):388-391.
4. Li X, Zhang F, Song G, Gu W, Chen M, Yang B, Li D, Wang D, Cao K: **Intramyocardial Injection of Pig Pluripotent Stem Cells Improves Left Ventricular Function and Perfusion: A Study in a Porcine Model of Acute Myocardial Infarction**. *PloS one* 2013, **8**(6):e66688.
5. Stuckey DJ, McSweeney SJ, Thin MZ, Habib J, Price AN, Fiedler LR, Gsell W, Prasad SK, Schneider MD: **T(1) mapping detects pharmacological retardation of diffuse cardiac fibrosis in mouse pressure-overload hypertrophy**. *Circ Cardiovasc Imaging* 2014, **7**(2):240-249.
