# Supplementary material for: Human Induced Pluripotent Stem Cell-Derived Cardiomyocyte Encapsulating Bioactive Hydrogels Improve Rat Heart Function Post Myocardial Infarction
Source: Stem Cell Reports. 2017 Oct 5;9(5):1415–22. doi: 10.1016/j.stemcr.2017.09.003 (PMC5830963; doi:10.1016/j.stemcr.2017.09.003)
Supplement: Document S2. Article plus Supplemental Information [file mmc2.pdf]

Stem Cell Reports
Report

OPEN ACCESS

Human Induced Pluripotent Stem Cell-Derived Cardiomyocyte Encapsulating Bioactive Hydrogels Improve Rat Heart Function Post Myocardial Infarction

Andre Chow,[1,7] Daniel J. Stuckey,[2,6,7] Emaddin Kidher,[1] Mark Rocco,[3,4,5] Richard J. Jabbour,[6] Catherine A. Mansfield,[6] Ara Darzi,[1] Sian E. Harding,[6,*] Molly M. Stevens,[3,4,5,*] and Thanos Athanasiou[1]
[1]Department of Surgery and Cancer, Imperial College London, St Mary's Hospital, London W2 1NY, UK
[2]Centre for Advanced Biomedical Imaging, University College London, London WC1E 6DD, UK
[3]Department of Materials, Faculty of Engineering, Imperial College London, South Kensington, London SW7 2AZ, UK
[4]Department of Bioengineering, Imperial College London, London SW7 2AZ, UK
[5]Institute for Biomedical Engineering, Imperial College London, London SW7 2AZ, UK
[6]National Heart & Lung Institute, Imperial College London, Hammersmith Campus, London W12 0NN, UK
[7]Co-first author
*Correspondence: sian.harding@imperial.ac.uk (S.E.H.), m.stevens@imperial.ac.uk (M.M.S.)
https://doi.org/10.1016/j.stemcr.2017.09.003

SUMMARY

Tissue engineering offers an exciting possibility for cardiac repair post myocardial infarction. We assessed the effects of combined polyethylene glycol hydrogel (PEG), human induced pluripotent stem cell-derived cardiomyocyte (iPSC-CM), and erythropoietin (EPO) therapy in a rat model of myocardial infarction. PEG with/out iPSC-CMs and EPO; iPSC-CMs in saline; or saline alone was injected into infarcted hearts shortly after infarction. Injection of almost any combination of the therapeutics limited acute elevations in chamber volumes. After 10 weeks, attenuation of ventricular remodeling was identified in all groups that received PEG injections, while ejection fractions were significantly increased in the gel-EPO, cell, and gel-cell-EPO groups. In all treatment groups, infarct thickness was increased and regions of muscle were identified within the scar. However, no grafted cells were detected. Hence, iPSC-CM-encapsulating bioactive hydrogel therapy can improve cardiac function post myocardial infarction and increase infarct thickness and muscle content despite a lack of sustained donor-cell engraftment.

INTRODUCTION

Pharmacological and surgical treatments have improved prognosis post myocardial infarction (MI), but there is still no effective method to replace the lost tissue, meaning that heart failure remains a leading cause of mortality and morbidity worldwide (Roger et al., 2012).

Regenerative medicine has the potential to repair damaged myocardium (Segers and Lee, 2008). Experimental studies show that grafting stem cells into the heart can directly replace the tissue damaged during MI (Orlic et al., 2001), while stimulation of innate repair mechanisms can activate resident progenitor cells and initiate regeneration (Ellison et al., 2013). Many cells with regenerative potential have been proposed, including those derived from blood, bone marrow, adipose, and cardiac tissue (Segers and Lee, 2008). Although each of these cell types has the ability to differentiate into new contractile cardiomyocytes, the efficiency of differentiation is low (Murry et al., 2006). Embryonic stem cells (ESCs) and induced pluripotent stem cells (iPSCs) have true cardiomyogenic potential (Zhang et al., 2009; Chong et al., 2014) and may offer a more effective cell type for cardiac regeneration. iPSCs can be expanded as highly purified and specific cell populations and avoid many of the immunological and ethical problems associated with ESCs (Yamanaka, 2009), making them suitable for clinical use (Chong et al., 2014).

A major current limitation of regenerative medicine is poor donor-cell retention. The most commonly used approach of directly injecting cells suspended in liquid solution into the infarcted heart results in the loss of 95%–99% of grafted cells within the first 24 hr (Sheikh et al., 2007). Tissue engineering can enhance cell retention and survival by delivering cells on or within biomaterials (Leor et al., 2005; Christman and Lee, 2006; Coulombe et al., 2014), including injectable hydrogels, tissue patches, cell sheets, or recellularized scaffolds (Jawad et al., 2008). These approaches can reduce immediate mechanical cell loss, provide a protective environment for cell survival, promote integration into the host tissue, and reduce myocardial wall stress by providing mechanical support for the damaged tissue (Place et al., 2009).

An advantage of hydrogels is the capacity for precise tailoring of mechanical properties and gelation times that allow them to be matched to the stiffness of the myocardium and delivery using current clinical catheter-based approaches, as opposed to the invasive procedures required for many other approaches (Hoffman, 2002; Frey et al., 2014). Synthetic hydrogels, including those based upon polyethylene glycol (PEG), have been used as a scaffold for drug and cell delivery (Lin and Anseth, 2009). They have excellent biocompatibility and safety records and are now well established for use in the medical field (Van

© 2017 The Author(s).
This is an open access article under the CC BY license (http://creativecommons.org/licenses/by/4.0/).

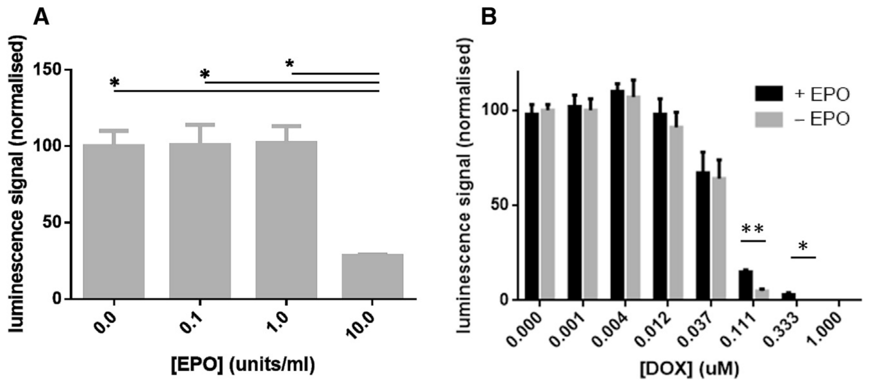

Figure 1. Effect of EPO on iPSC-CM Viability

(A) EPO dose response for iPSC-CMs *in vitro*. Increasing concentrations of EPO were added to iPSC-CM cultures for 6 hr (n = 4 individual replicates for each group). Cell viability was unaffected at 0.1 and 1 units/mL but compromised at 10 units/mL.

(B) Effect of 0.1 units/mL EPO on doxorubicin (DOX)-induced injury of iPSC-CMs. iPSC-CM cultures were exposed to increasing concentrations of doxorubicin for 6 hr with or without the presence of 1 unit/mL EPO. Cell viability was greater in the presence of EPO.

Data are presented as mean ± SD. $^{**}p < 0.01$, $^{*}p < 0.05$.

Tomme et al., 2008). In addition, bioactive agents, such as growth factors, hormones, and small molecules, can easily be incorporated within the matrix. These can enhance donor-cell survival, integration, proliferation, and differentiation and modulate the host's immune and innate regenerative response (Laflamme et al., 2007). One such agent, erythropoietin (EPO), has already been successful in the clinic for reduction of cell death and remodeling post MI (Brines and Cerami, 2008) and has shown benefits in experimental infarction when delivered using a gelatin cardiac patch (Kobayashi et al., 2008).

The aim of the current study was to determine whether combining mechanically tailored injectable hydrogels, iPSC therapy, and the cardioprotective molecule EPO could provide a novel strategy to prevent cardiac failure in a rat model of MI.

RESULTS

Hydrogels Can Be Tailored to the Properties Desired for Cardiac Repair

Hydrogels were formed by copolymerization of 4-arm PEG acrylate with PEG dithiol in stoichiometrically balanced ratios. Rheometry analysis of the evolution of storage modulus G′ and loss modulus G″ was used to calculate the gelation times for 5%, 10%, 20%, and 30% w/v hydrogels (n = 4) as 352 ± 123, 195 ± 42, 140 ± 14, and 138 ± 29 s, respectively. Shear modulus (maximum G′) for 5%, 10%, 20%, and 30% hydrogels was 0.8 ± 0.3, 6.9 ± 1.8, 17.2 ± 1.2, and 34.9 ± 1.2 kPa, respectively. Wet weight measurements indicated that the gel degradation rate decreased with increased hydrogel percentage, with a time to total degradation of 8, 16, 21, and 22 days for 5%, 10%, 20%, and 30% hydrogels (n = 4), respectively (Figure S2). The optimal hydrogel would have a gelation time of around 180 s to permit catheter-based delivery with rapid cross-linking once *in situ*; a calculated shear modulus between normal (6 kPa) and infarcted (18 kPa) myocardium (Berry et al., 2006); and a total degradation time greater than 10 days to allow donor-cell integration. The 10% PEG hydrogel most closely matched these parameters and was used in all later experiments.

EPO Protects Against Oxidative Stress and Cell Death *In Vitro*

The iPSC-cardiomyocytes (iPSC-CMs) used in these studies were purchased from Cellular Dynamics Inc. and offer a highly purified cardiomyocyte cell line in which 98.2 ± 1.1% of cells expressed the cardiac-specific marker α-major histocompatibility complex (MHC). To assess the effects of EPO, iPSC-CMs were exposed to 0.1 to 10 units/mL EPO. Cell viability was unaltered up to 1 unit/mL EPO but was significantly reduced by 10 units/mL EPO (Figure 1A). To assess whether EPO was cardioprotective under cell-stress conditions, iPSC-CMs were exposed to doxorubicin. Cell viability was reduced in the presence of doxorubicin. However, addition of 1 unit/mL EPO significantly increased cell viability (Figure 1B). This suggests that EPO has therapeutic potential for use in MI treatment and in tandem with cell therapy.

Hydrogel Injection Does Not Inhibit Normal Heart Function

To test potentially deleterious effects of biomaterial injection, PEG hydrogels were injected into Langendorff perfused hearts *ex vivo* (n = 5) and naive rat hearts *in vivo* (n = 5). No arrhythmia or significant change in heart rate or left ventricular (LV) pressure was observed in the Langendorff preparation, with heart rate 102% and mean LV pressure 93% of pre-injection values. No alteration to *in vivo* cardiac function was identified using cardiac magnetic resonance imaging (MRI), with end

Table 1. *In Vivo* and Histological Assessment of Cardiac Structure, Function, and Viability

		Sham n = 4/4	MI n = 8/7	Gel n = 8/6	Gel-EPO n = 6/5	Cell n = 9/7	Gel-Cell-EPO n = 8/5	ANOVA
End diastolic volume (μL)	acute	290 ± 45	407 ± 51[a]	348 ± 66	324 ± 49	328 ± 72	327 ± 39	0.018
	chronic	422 ± 36	632 ± 65[a]	598 ± 89	565 ± 146	649 ± 155[a]	592 ± 60	0.038
End systolic volume (μL)	acute	88 ± 15	222 ± 44[a]	194 ± 65[a]	151 ± 62	165 ± 55	181 ± 34	0.003
	chronic	101 ± 10	334 ± 54[a]	283 ± 85[a]	220 ± 135	298 ± 103[a]	272 ± 77	0.005
Late gadolinium enhancement (%)	acute	3.2 ± 1.7	36.2 ± 7.3[a]	38.3 ± 8.0[a]	29.1 ± 9.3[a]	38.4 ± 12.5[a]	37.0 ± 8.1[a]	<0.001
	chronic	1.4 ± 1.1	27.1 ± 4.4[a]	28.3 ± 4.4[a]	16.7 ± 7.2[a,b]	21.1 ± 5.1[a]	21.6 ± 2.6[a]	<0.001
Ejection fraction (%)	acute	69.8 ± 1.1	45.4 ± 7.8[a]	45.2 ± 10.2[a]	54.6 ± 12.6	50.6 ± 8.9[a]	44.8 ± 8.4[a]	<0.001
	chronic	76.2 ± 0.9	47.3 ± 6.1[a]	53.1 ± 10.5[a]	63.5 ± 11.6[b,d]	55.3 ± 8.0[a,d]	54.7 ± 8.1[a,d]	<0.001
Infarct thickness (mm)	chronic	–	1.34 ± 0.27	1.99 ± 0.34[b]	1.66 ± 0.37[b]	1.92 ± 0.35[b]	1.98 ± 0.41[b]	<0.001
Infarct muscle content (%)	chronic	–	9.2 ± 3.3	22.7 ± 10.1[b]	18.1 ± 10.5	24.2 ± 3.4[b]	36.4 ± 6.3[b,c]	<0.001

A one-way between-subjects ANOVA test to compare the values between all groups. Post hoc analysis (Tukey's test), statistically significant ($p < 0.05$) difference between group versus sham ([a]); group versus MI ([b]); group versus all others ([c]). Student's t test analysis, statistically significant ($p < 0.05$) difference between acute (<48 hr) and chronic (10 weeks) time points ([d]).

diastolic volume (EDV) of 412 ± 24 μL before and 400 ± 54 μL after and ejection fraction (EF) of 73% ± 5% before and 78% ± 11% after.

Bioactive Hydrogel Injections Improve Heart Function Post MI

Forty-three male nude rats survived MI or sham surgery and underwent cardiac MRI within 48 hr. As expected, EF was lower and infarct size higher in all infarcted groups. Injection of almost any combination of gel, cells, or EPO slightly reduced the expected increase in EDV and ESV at this early time point (Table 1 and Figure 2).

Nine rats did not survive until the 10 week MRI scan. This level of death is expected in this model and was not specific to any treatment group. At 10 weeks, EDV in saline and cell-alone treated hearts was increased compared with shams. However, EDVs were not significantly higher than shams in animals that received any form of gel injection, suggesting reduction of LV remodeling in groups that received hydrogel. EF significantly increased from the acute to the chronic MRI time points in the gel-EPO, cell, and gel-cell-EPO groups, although not in the control MI and gel groups. Percentage infarct sizes reduced in all groups owing to thinning of the infarcted tissue and hypertrophy of viable myocardium (Table 1 and Figure 2). Regional analysis of contrast-enhanced MRI demonstrated increased muscle mass within the infarcted apical region of the left ventricle, suggesting that functional improvements may have been due to greater viable muscle within the infarcted regions (Figure S3).

Therapies Increase Infarct Thickness and Muscle Content but Not Through Donor-Cell Engraftment

At 10 weeks, Masson's trichrome staining demonstrated that the thickness of the infarcted region was greater in all treated groups compared with control MI hearts (Figure 3C). Further, the percentage of muscle content within the infarcted region was also greater in every group except gel-EPO, suggesting that some form of tissue salvage or regeneration had occurred (Figure 3D). Muscle content within the infarcted region was greatest in the gel-cell-EPO group and significantly enhanced in comparison with any other treatment group ($p < 0.005$).

However, immunohistochemical staining for human cardiac troponin T within iPSC-CM grafted rat tissue was unable to identify any engrafted human cells at 10 weeks after injection. In addition, qPCR for expression of known early and late cardiac markers, including atrial natriuretic factor (*NPPA*), myosin-6 (*MYH6*), myosin-7 (*MYH7*), and alpha-1A and 1B adrenergic receptors (*ADRA1a* and *ADRA1b*) was unable to identify any human gene transcripts within the rat tissue. Staining and qPCR for human cells performed on a subset of rat hearts taken shortly after iPSC-CM injection was able to detect grafted human cells (Figure S4).

DISCUSSION

Determining the optimal and most clinically relevant methods for enhancing the potential of stem cell-mediated

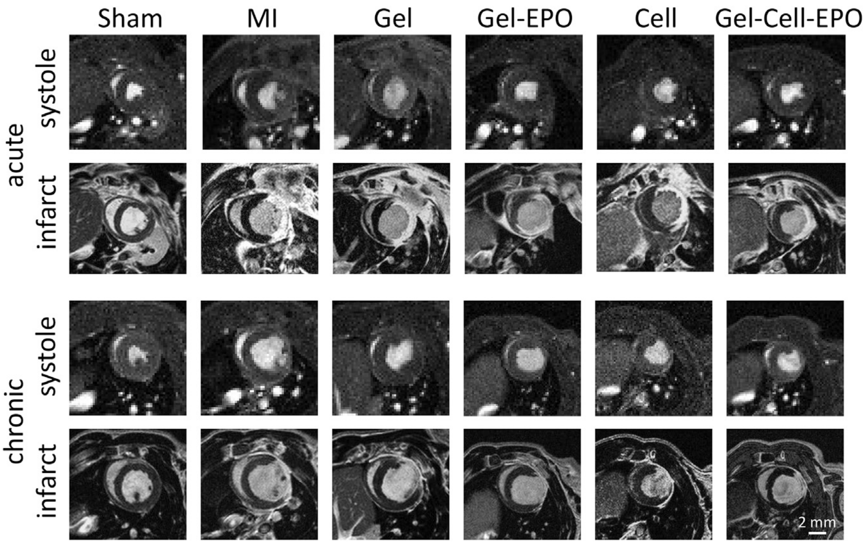

Figure 2. MRI Characterization of Cardiac Structure, Function, and Viability Acutely and Chronically after MI
The systolic image shows the end systolic frame of a mid-ventricular cine-MR image. The infarct image shows a late gadolinium-enhanced MRI of the hyper-enhanced infarct region at the same position as the cine-MR image.

cardiac repair is essential for the future of regenerative medicine. We compared the efficacy of clinically applicable biomaterials, biologics, and human iPSC-CMs in a rat model of MI using state-of-the-art pre-clinical cardiac MRI.

Although many cell types with potential to repair the damaged heart have been identified, the challenge of efficiently delivering, protecting, and nurturing them within the hostile post-MI environment has not been met. Tissue engineering is being intensely investigated as a way to address this problem, but such approaches must use clinically acceptable materials, delivery approaches, biologics, and cell types. Here, we engineered a clinically approved PEG hydrogel to have gelation time comparable with catheter-based delivery, shear modulus similar to the host myocardium, and a degradation profile that would support donor-cell integration; all properties desirable for cardiac repair.

Hydrogel injections did not disrupt normal heart function, with no observed arrhythmia or alterations to heart rate, mean LV pressure, or cardiac function. Injection of the hydrogel into the myocardium acutely post MI tempered the expected early increase in EDV and ESV seen in infarcted hearts. This suggests that the compliance of the biomaterial is suited to reducing wall stress acutely post MI and can limit initial chamber dilation. At 10 weeks, EDV was not significantly greater than sham-operated animals in any of the groups that received a gel injection, while control MI and cell-injected MI hearts had increased EDV. This provides further evidence that a 10% PEG hydrogel can limit LV remodeling post MI. These results support a previous *in silico* study predicting that the injection of non-contractile biomaterials into a finite element model simulation of the infarcted heart would reduce EDV (Wall et al., 2006). In addition, acellular alginate-based hydrogels have been tested clinically in MI patients and show preservation of LV indices (Frey et al., 2014). Taken together with the data presented here, these results support the use of injectable acellular biomaterials to reduce detrimental remodeling post MI.

Delivery of any therapy did not significantly alter the acute late gadolinium-enhanced (LGE) MRI infarct size compared with control MIs, suggesting therapeutic benefits were not directly related to early myocardial salvage. Between 48 hr and 10 weeks, EF significantly increased in the gel-EPO, cell, and gel-cell-EPO groups but not in the control MI or gel groups. This suggests that incorporation of a biological component within the gel is essential for an active increase in cardiac function, as opposed to the passive restriction of remodeling that occurred in the gel-alone group where EDV was reduced but EF was not enhanced. The degree of EF enhancement at the time points studied was similar across the gel-EPO, cell, gel-cell-EPO groups, suggesting that the mechanism of improved contraction was not cumulative or directly linked to a single component of the regenerative platform.

There have been previous reports of a tissue engineering approach combining biomaterials, soluble factors, and cell therapy for cardiac failure. In a rat model of MI, Davis et al. (2006) demonstrated a significant improvement in function in a combined nanofiber, insulin-like growth factor 1, and neonatal cardiomyocyte therapy group after 3 weeks, implying that the combination of all three therapies was required for significant benefit. Takehara et al. (2008) used a porcine model of MI to investigate the combination of a gelatin hydrogel, basic fibroblast growth factor (bFGF), and human cardiosphere-derived cells (hCDCs). After 4 weeks, they demonstrated a synergistic effect of hCDCs and the bFGF-loaded hydrogel upon EF and infarct volume.

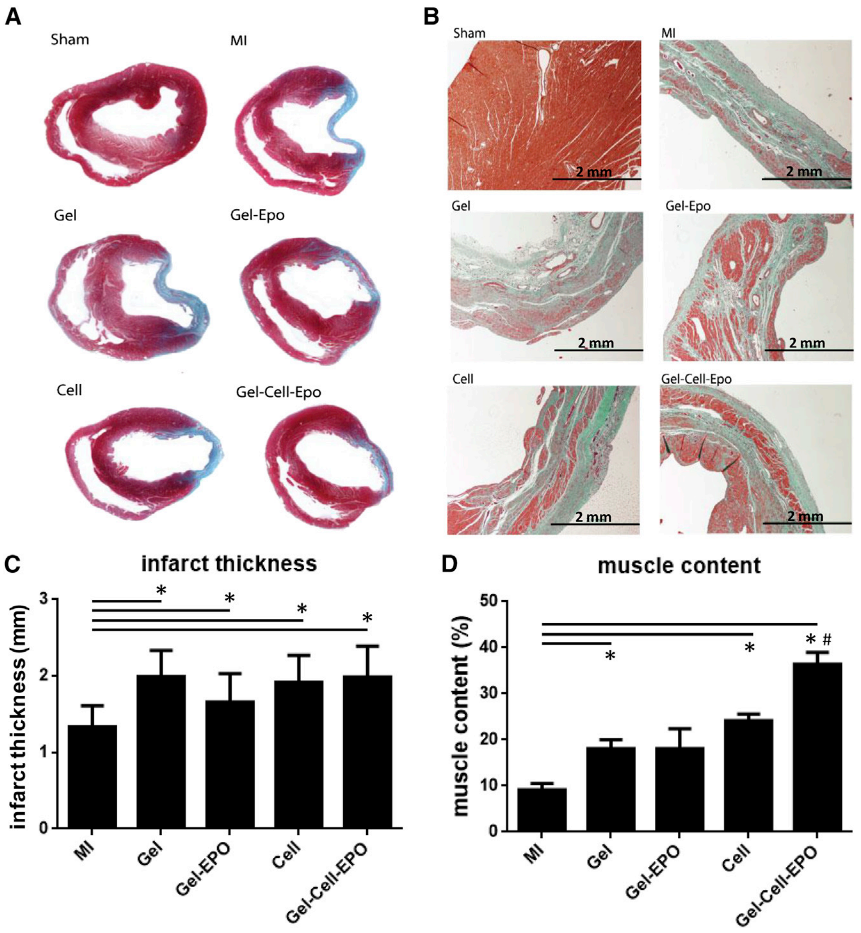

Figure 3. Masson's Trichrome Staining of Infarct Size

(A) Masson's trichrome staining of mid-ventricular cardiac sections 10 weeks after infarction. Collagen fibers stain light blue and muscle fibers stain red.

(B) Magnified views (×4) show muscle fibers (red) within the collagen-rich scar (blue).

(C) Infarct thickness of hearts stained with Masson's trichrome stain after the chronic imaging time point. Each therapy increased the thickness of the infarcted tissue.

(D) The proportion of the infarct scar containing muscle fibers in the Masson's stained heart sections shows that all therapies, except gel-EPO, increased the viable tissue content within the infarct.

Data are presented as mean ± SD. Statistically significant difference between group versus MI(* $p < 0.05$); group versus all other groups (# $p < 0.005$).

More recently, Kraehenbuehl et al. (2010) utilized a combination of a matrix-metalloproteinase-sensitive PEG hydrogel, thymosin β4, and human ESC-derived vascular cells in a rat model of MI. Using cardiac MRI, they demonstrated significant effects after 6 weeks upon infarct size, EDV, and EF, which was most pronounced in the combined hydrogel-thymosin β4-cell therapy group. The current study is the first to combine PEG hydrogels with iPSC-CMs and EPO. Rapid translation of this combination therapy to human trials is feasible as PEG-based compounds are already in extensive use in human medicine (Lin and Anseth, 2009), and EPO has already demonstrated safety and efficacy in the treatment of other human diseases (Coleman and Brines, 2004).

At 10 weeks, the thickness of the infarct in histological sections was increased in all treated groups. Further analysis of the extent of muscle fibers within the collagenous infarct zone revealed muscle salvage or regeneration was greater in the gel, cell, and gel-cell-EPO groups than control MI, and that gel-cell-EPO injection resulted in significantly more infarct muscle content than any other treatment. This suggests that the combination gel-cell-EPO therapy is optimal for salvaging or regenerating the infarct. Although gel-cell-EPO therapy did not lead to greater functional improvement *in vivo* after 10 weeks than that seen with other groups, it might be anticipated that benefits in function and survival would become apparent in more chronic post-MI remodeling. The possibility that the observed benefits resulted directly from PEG-mediated enhancement of cell retention cannot be answered by this study as a gel-cell group was not included. Likewise, the omission of a cell-EPO group precludes further discussion of the benefits of EPO on cell retention and resultant myocardial viability.

Importantly, immunohistochemistry and qPCR (methods that are able to detect cell grafts acutely after injection; Figure S4) did not detect any cells of human origin within the grafted hearts at 10 weeks. This indicates that the muscle fibers identified within the infarct region of treated hearts were not derived from integration of the grafted human iPSC-CMs but likely signifies either salvaged myocardium protected from cell death during acute MI, regenerated myocardium derived from the host endogenous stem cells (Ellison et al., 2013), or enhanced tissue

vascularity, although unfortunately these processes were not measured in this study. It is disappointing that the advanced biomaterials approach employed here was unable to facilitate stable engraftment of iPSC-CMs within the host tissue, especially as we have previously shown enhanced cell retention within the myocardium using PEG hydrogels (Speidel et al., 2017). However, it is encouraging that the strategy did lead to increased cardiac function and infarct muscle content.

The use of cardiac MRI adds accuracy and clinical relevance to this study, as similar non-invasive assessments of function and tissue viability are employed in leading clinical trials of cell therapy for the heart (Bolli et al., 2011; Makkar et al., 2012). The ability to measure acute infarct size *in vivo* using LGE MRI can ensure that surgically induced infarct sizes are similar shortly after infarction, and that subsequent changes in function are due to therapy, rather than variation in initial infarct size. In this study, LGE infarct size was similar acutely after MI in the MI, gel, cell, gel-cell-EPO groups. However, the gel-EPO group had smaller LGE infarct size acutely after MI. This could be the result of variability in surgical procedure or might indicate that EPO can limit the degree of acute cell death during MI, as has previously been reported by others (Brines and Cerami, 2008; Robey et al., 2008).

A wide variety of cell types and biologics have been shown to initiate improvements in cardiac function post MI, both in the pre-clinical setting and in humans (Sanganalmath and Bolli, 2013). However, it is frequently reported that the number of grafted cells that remain within the heart is minimal at later time points (Gnecchi et al., 2005; Passier et al., 2008; Carr et al., 2011; Kawamura et al., 2012; Noseda et al., 2015). Taken in context, the data presented here indicate that the beneficial effects of grafting this bioactive hydrogel derive from a paracrine mechanism, with gel, cell, and EPO delivery initiating protective pathways that reduce cell death and stimulate endogenous repair, resulting in increased contractility and muscle content in the absence of sustained engraftment of donor cells.

EXPERIMENTAL PROCEDURES

Expanded methods are available in the Supplemental Information.

Hydrogel Preparation

PEG hydrogels were formed using a Michael addition reaction by adding PEG dithiol (molecular weight = 1,019; Sigma-Aldrich, Dorset, UK) dissolved in 1% triethanolamine to 4-arm PEG acrylate (molecular weight = 23,300; Arab, AL, USA) dissolved in PBS. Viscoelastic properties were assessed using a rheometer (AR 2000ex; TA Instruments, Crawley, UK) at 37°C. Gel properties were examined by frequency sweep experiments at 1–10 Hz (ω = 6.3–62.83 rad/s) at a fixed strain amplitude of 0.001 (0.1%) in order to ensure uniform mechanical properties were maintained at frequencies present in human (1 Hz, 6.3 rad/s) and rat (8 Hz, 2.3 rad/s) heart.

Cell Culture

iPSC-CMs (Cellular Dynamics Inc, Madison, WI, USA) were thawed and plated onto 0.1% gelatin-coated surfaces at a density of ~55,000 cells per cm^2 for 1 week prior to injection. 98.2% ± 1.1% of iPSC-CMs expressed α-MHC, and viability was not significantly affected when cells were passed through the injection needle as determined using an automated cell counter (Luna-FL, Logos Biosystems) and acridine orange/propidium iodide staining.

Animal Model of Myocardial Infarction

All animal experiments were conducted under Home Office Project License 70/6568 and in accordance with local the Imperial College Ethics Committee and the ARRIVE Guidelines. The study design is illustrated in Figure S1. Eight-week old male nude rats (Charles River, Germany) underwent MI by occlusion of the left anterior descending artery as described (Carr et al., 2011). Sham surgery was performed by passing a suture and needle through the myocardium without ligation.

Injection of Therapeutics

A 10% 4-arm PEG acrylate solution was prepared by 1:1 mixing of 20% 4-arm PEG acrylate solution with cell suspension, and the appropriate volume of 10% PEG dithiol was added to this mixture just prior to injection to initiate the gelling process. Hydrogels (3 × 50 μL), cells (5 × 10^5), or a hydrogel and cell mixture was injected around the infarct border zone using a 27G needle. The experimental groups were as follows: sham, sham-operated control group that received PBS injection; MI, myocardial infarction group that received PBS injection; gel, MI group that received 10% PEG hydrogel injection; gel-EPO, MI group that received 10% PEG hydrogel containing 1 unit/mL EPO; cell, MI group that received 5 × 10^5 iPSC-CMs in PBS; gel-cell-EPO, MI group that received 10% PEG hydrogel containing 1 unit/mL EPO and 5 × 10^5 iPSC-CMs.

Cardiac MRI

Cardiac MRI was performed at 48 hr and 10 weeks as described (Stuckey et al., 2014) in anesthetized rats using a 4.7 T DirectDrive Varian MRI System (Palo Alto, CA, USA). Cardiac and respiratory-gated cine-MRI was used to measure cardiac structure and function. Infarct size was assessed by LGE MRI performed 25 min after intraperitoneal injection of 0.5 mg/kg Gd-DTPA-BMA (Omniscan) using a multi-slice inversion recovery sequence (Stuckey et al., 2014). Data were analyzed using ImageJ. MRI acquisition and analysis were performed by an investigator blinded to the experimental groups.

Histology and qPCR

Ten weeks after infarction, the animals were killed; hearts were fixed and cryosectioned for immunostaining or Masson's trichrome staining. RNA was extracted (RNeasy Mini Kit, QIAGEN) and qPCR performed in triplicate using pre-validated TaqMan assays (Life Technologies) for *NPPA*, *MYH6*, *MYH7*, and *ADRA1a* and *b*.

Statistical Analysis

Using GraphPad Prism, one-way between groups ANOVA($\alpha = 0.05$) was performed for group comparison. If the results of the ANOVA were found to be significant, post hoc analysis was performed using the Tukey multiple comparisons test to compare results among all groups.

SUPPLEMENTAL INFORMATION

Supplemental Information includes Supplemental Experimental Procedures and four figures and can be found with this article online at https://doi.org/10.1016/j.stemcr.2017.09.003.

AUTHOR CONTRIBUTIONS

A.C., D.J.S., A.D., S.E.H., M.M.S., and T.A. planned and designed experiments. A.C., D.J.S., M.R., R.J.J., and C.A.M. performed and analyzed experiments. A.C., D.J.S., E.K., S.E.H., M.M.S., and T.A. wrote the manuscript.

ACKNOWLEDGMENTS

The research was supported by the National Institute for Health Research (NIHR) Biomedical Research Centre based at Imperial College Healthcare NHS Trust and Imperial College London and grants from the BHF Regenerative Medicine Centre (RM/13/1/30157) and The Wellcome Trust Clinical PhD Fellowship scheme. D.J.S. is a BHF Intermediate Basic Science Research Fellow (FS/15/33/31608). M.M.S. is supported by a Wellcome Trust Senior Investigator Award (098411/Z/12/Z). Raw data is available upon request from rdm-enquiries@imperial.ac.uk.

Received: November 13, 2016
Revised: September 1, 2017
Accepted: September 1, 2017
Published: October 5, 2017

REFERENCES

Berry, M.F., Engler, A.J., Woo, Y.J., Pirolli, T.J., Bish, L.T., Jayasankar, V., Morine, K.J., Gardner, T.J., Discher, D.E., and Sweeney, H.L. (2006). Mesenchymal stem cell injection after myocardial infarction improves myocardial compliance. Am. J. Physiol. Heart Circ. Physiol. *290*, H2196–H2203.

Bolli, R., Chugh, A.R., D'Amario, D., Loughran, J.H., Stoddard, M.F., Ikram, S., Beache, G.M., Wagner, S.G., Leri, A., Hosoda, T., et al. (2011). Cardiac stem cells in patients with ischaemic cardiomyopathy (SCIPIO): initial results of a randomised phase 1 trial. Lancet *378*, 1847–1857.

Brines, M., and Cerami, A. (2008). Erythropoietin-mediated tissue protection: reducing collateral damage from the primary injury response. J. Intern. Med. *264*, 405–432.

Carr, C.A., Stuckey, D.J., Tan, J.J., Tan, S.C., Gomes, R.S., Camelliti, P., Messina, E., Giacomello, A., Ellison, G.M., and Clarke, K. (2011). Cardiosphere-derived cells improve function in the infarcted rat heart for at least 16 weeks–an MRI study. PLoS One *6*, e25669.

Chong, J.J., Yang, X., Don, C.W., Minami, E., Liu, Y.W., Weyers, J.J., Mahoney, W.M., Van Biber, B., Cook, S.M., Palpant, N.J., et al. (2014). Human embryonic-stem-cell-derived cardiomyocytes regenerate non-human primate hearts. Nature *510*, 273–277.

Christman, K.L., and Lee, R.J. (2006). Biomaterials for the treatment of myocardial infarction. J. Am. Coll. Cardiol. *48*, 907–913.

Coleman, T., and Brines, M. (2004). Science review: recombinant human erythropoietin in critical illness: a role beyond anemia? Crit. Care *8*, 337–341.

Coulombe, K.L., Bajpai, V.K., Andreadis, S.T., and Murry, C.E. (2014). Heart regeneration with engineered myocardial tissue. Annu. Rev. Biomed. Eng. *16*, 1–28.

Davis, M.E., Hsieh, P.C., Takahashi, T., Song, Q., Zhang, S., Kamm, R.D., Grodzinsky, A.J., Anversa, P., and Lee, R.T. (2006). Local myocardial insulin-like growth factor 1 (IGF-1) delivery with biotinylated peptide nanofibers improves cell therapy for myocardial infarction. Proc. Natl. Acad. Sci. USA *103*, 8155–8160.

Ellison, G.M., Vicinanza, C., Smith, A.J., Aquila, I., Leone, A., Waring, C.D., Henning, B.J., Stirparo, G.G., Papait, R., Scarfo, M., et al. (2013). Adult c-kit(pos) cardiac stem cells are necessary and sufficient for functional cardiac regeneration and repair. Cell *154*, 827–842.

Frey, N., Linke, A., Suselbeck, T., Muller-Ehmsen, J., Vermeersch, P., Schoors, D., Rosenberg, M., Bea, F., Tuvia, S., and Leor, J. (2014). Intracoronary delivery of injectable bioabsorbable scaffold (IK-5001) to treat left ventricular remodeling after ST-elevation myocardial infarction: a first-in-man study. Circ. Cardiovasc. Interv. *7*, 806–812.

Gnecchi, M., He, H., Liang, O.D., Melo, L.G., Morello, F., Mu, H., Noiseux, N., Zhang, L., Pratt, R.E., Ingwall, J.S., and Dzau, V.J. (2005). Paracrine action accounts for marked protection of ischemic heart by Akt-modified mesenchymal stem cells. Nat. Med. *11*, 367–368.

Hoffman, A.S. (2002). Hydrogels for biomedical applications. Adv. Drug Deliv. Rev. *54*, 3–12.

Jawad, H., Lyon, A.R., Harding, S.E., Ali, N.N., and Boccaccini, A.R. (2008). Myocardial tissue engineering. Br. Med. Bull. *87*, 31–47.

Kawamura, M., Miyagawa, S., Miki, K., Saito, A., Fukushima, S., Higuchi, T., Kawamura, T., Kuratani, T., Daimon, T., Shimizu, T., et al. (2012). Feasibility, safety, and therapeutic efficacy of human induced pluripotent stem cell-derived cardiomyocyte sheets in a porcine ischemic cardiomyopathy model. Circulation *126* (11 Suppl 1), S29–S37.

Kobayashi, H., Minatoguchi, S., Yasuda, S., Bao, N., Kawamura, I., Iwasa, M., Yamaki, T., Sumi, S., Misao, Y., Ushikoshi, H., et al. (2008). Post-infarct treatment with an erythropoietin-gelatin hydrogel drug delivery system for cardiac repair. Cardiovasc. Res. *79*, 611–620.

Kraehenbuehl, T.P., Ferreira, L.S., Hayward, A.M., Nahrendorf, M., van der Vlies, A.J., Vasile, E., Weissleder, R., Langer, R., and Hubbell, J.A. (2010). Human embryonic stem cell-derived microvascular grafts for cardiac tissue preservation after myocardial infarction. Biomaterials *32*, 1102–1109.

Laflamme, M.A., Chen, K.Y., Naumova, A.V., Muskheli, V., Fugate, J.A., Dupras, S.K., Reinecke, H., Xu, C., Hassanipour, M., Police, S., et al. (2007). Cardiomyocytes derived from human embryonic

stem cells in pro-survival factors enhance function of infarcted rat hearts. Nat. Biotechnol. *25*, 1015–1024.

Leor, J., Amsalem, Y., and Cohen, S. (2005). Cells, scaffolds, and molecules for myocardial tissue engineering. Pharmacol. Ther. *105*, 151–163.

Lin, C.C., and Anseth, K.S. (2009). PEG hydrogels for the controlled release of biomolecules in regenerative medicine. Pharm. Res. *26*, 631–643.

Makkar, R.R., Smith, R.R., Cheng, K., Malliaras, K., Thomson, L.E., Berman, D., Czer, L.S., Marban, L., Mendizabal, A., Johnston, P.V., et al. (2012). Intracoronary cardiosphere-derived cells for heart regeneration after myocardial infarction (CADUCEUS): a prospective, randomised phase 1 trial. Lancet *379*, 895–904.

Murry, C.E., Reinecke, H., and Pabon, L.M. (2006). Regeneration gaps: observations on stem cells and cardiac repair. J. Am. Coll. Cardiol. *47*, 1777–1785.

Noseda, M., Harada, M., McSweeney, S., Leja, T., Belian, E., Stuckey, D.J., Abreu Paiva, M.S., Habib, J., Macaulay, I., de Smith, A.J., et al. (2015). PDGFRalpha demarcates the cardiogenic clonogenic Sca1+ stem/progenitor cell in adult murine myocardium. Nat. Commun. *6*, 6930.

Orlic, D., Kajstura, J., Chimenti, S., Jakoniuk, I., Anderson, S.M., Li, B., Pickel, J., McKay, R., Nadal-Ginard, B., Bodine, D.M., et al. (2001). Bone marrow cells regenerate infarcted myocardium. Nature *410*, 701–705.

Passier, R., van Laake, L.W., and Mummery, C.L. (2008). Stem-cell-based therapy and lessons from the heart. Nature *453*, 322–329.

Place, E.S., Evans, N.D., and Stevens, M.M. (2009). Complexity in biomaterials for tissue engineering. Nat. Mater. *8*, 457–470.

Robey, T.E., Saiget, M.K., Reinecke, H., and Murry, C.E. (2008). Systems approaches to preventing transplanted cell death in cardiac repair. J. Mol. Cell. Cardiol. *45*, 567–581.

Roger, V.L., Go, A.S., Lloyd-Jones, D.M., Benjamin, E.J., Berry, J.D., Borden, W.B., Bravata, D.M., Dai, S., Ford, E.S., Fox, C.S., et al. (2012). Heart disease and stroke statistics–2012 update: a report from the American Heart Association. Circulation *125*, e2–e220.

Sanganalmath, S.K., and Bolli, R. (2013). Cell therapy for heart failure: a comprehensive overview of experimental and clinical studies, current challenges, and future directions. Circ. Res. *113*, 810–834.

Segers, V.F., and Lee, R.T. (2008). Stem-cell therapy for cardiac disease. Nature *451*, 937–942.

Sheikh, A.Y., Lin, S.A., Cao, F., Cao, Y., van der Bogt, K.E., Chu, P., Chang, C.P., Contag, C.H., Robbins, R.C., and Wu, J.C. (2007). Molecular imaging of bone marrow mononuclear cell homing and engraftment in ischemic myocardium. Stem Cells *25*, 2677–2684.

Speidel, A.T., Stuckey, D.J., Chow, L.W., Jackson, L.H., Noseda, M., Abreu Paiva, M., Schneider, M.D., and Stevens, M.M. (2017). Multimodal hydrogel-based platform to deliver and monitor cardiac progenitor/stem cell engraftment. ACS Cent. Sci. *3*, 338–348.

Stuckey, D.J., McSweeney, S.J., Thin, M.Z., Habib, J., Price, A.N., Fiedler, L.R., Gsell, W., Prasad, S.K., and Schneider, M.D. (2014). T(1) mapping detects pharmacological retardation of diffuse cardiac fibrosis in mouse pressure-overload hypertrophy. Circ. Cardiovasc. Imaging *7*, 240–249.

Takehara, N., Tsutsumi, Y., Tateishi, K., Ogata, T., Tanaka, H., Ueyama, T., Takahashi, T., Takamatsu, T., Fukushima, M., Komeda, M., et al. (2008). Controlled delivery of basic fibroblast growth factor promotes human cardiosphere-derived cell engraftment to enhance cardiac repair for chronic myocardial infarction. J. Am. Coll. Cardiol. *52*, 1858–1865.

Van Tomme, S.R., Storm, G., and Hennink, W.E. (2008). In situ gelling hydrogels for pharmaceutical and biomedical applications. Int. J. Pharm. *355*, 1–18.

Wall, S.T., Walker, J.C., Healy, K.E., Ratcliffe, M.B., and Guccione, J.M. (2006). Theoretical impact of the injection of material into the myocardium: a finite element model simulation. Circulation *114*, 2627–2635.

Yamanaka, S. (2009). A fresh look at iPS cells. Cell *137*, 13–17.

Zhang, J., Wilson, G.F., Soerens, A.G., Koonce, C.H., Yu, J., Palecek, S.P., Thomson, J.A., and Kamp, T.J. (2009). Functional cardiomyocytes derived from human induced pluripotent stem cells. Circ. Res. *104*, e30–41.

Stem Cell Reports, Volume *9*

Supplemental Information

Human Induced Pluripotent Stem Cell-Derived Cardiomyocyte Encapsulating Bioactive Hydrogels Improve Rat Heart Function Post Myocardial Infarction

Andre Chow, Daniel J. Stuckey, Emaddin Kidher, Mark Rocco, Richard J. Jabbour, Catherine A. Mansfield, Ara Darzi, Sian E. Harding, Molly M. Stevens, and Thanos Athanasiou

Figure S1

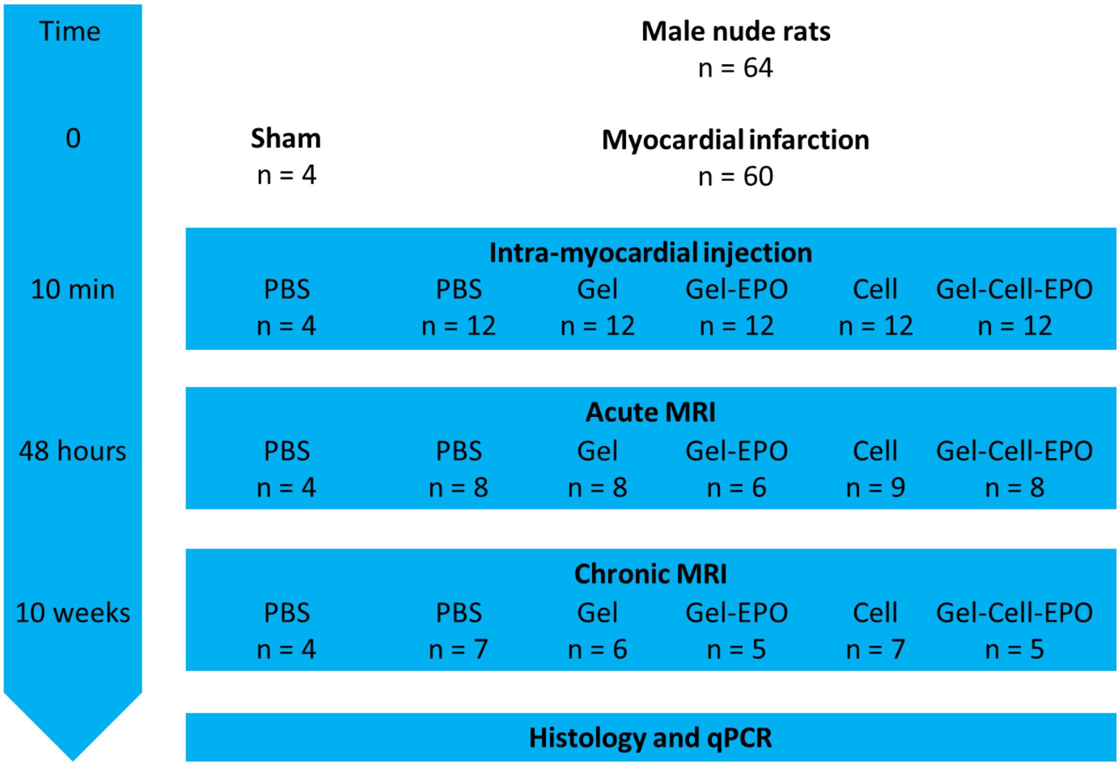
Time
0
10 min
48 hours
10 weeks
Male nude rats
n = 64
Sham
n = 4
Myocardial infarction
n = 60
Intra-myocardial injection
PBS n = 4
PBS n = 12
Gel n = 12
Gel-EPO n = 12
Cell n = 12
Gel-Cell-EPO n = 12
Acute MRI
PBS n = 4
PBS n = 8
Gel n = 8
Gel-EPO n = 6
Cell n = 9
Gel-Cell-EPO n = 8
Chronic MRI
PBS n = 4
PBS n = 7
Gel n = 6
Gel-EPO n = 5
Cell n = 7
Gel-Cell-EPO n = 5
Histology and qPCR

Figure S2

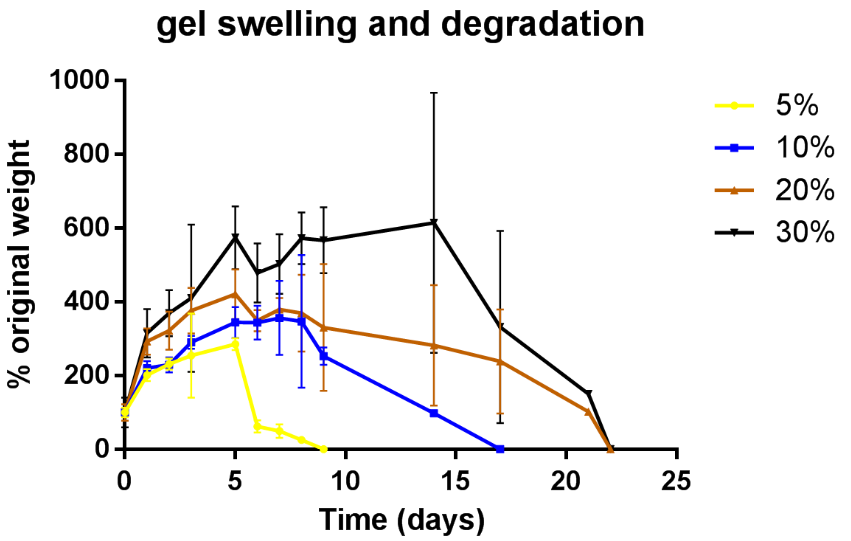

Figure S2. Hydrogel swelling and degradation

300 µl of PEG hydrogels (PEG4A + PEGDT) were formed at 5%, 10%, 20% and 30% (n = 4 individual replicates for each group), set overnight and immersed in 1ml of PBS. Wet weight of the gels was measured up to 22 days and expressed as a percentage of the original weight. Hydrogels of higher weight/volume concentrations swelled to a greater extent and took longer to degrade than lower concentration hydrogels. Data are presented as mean ± S.D.

Figure S3

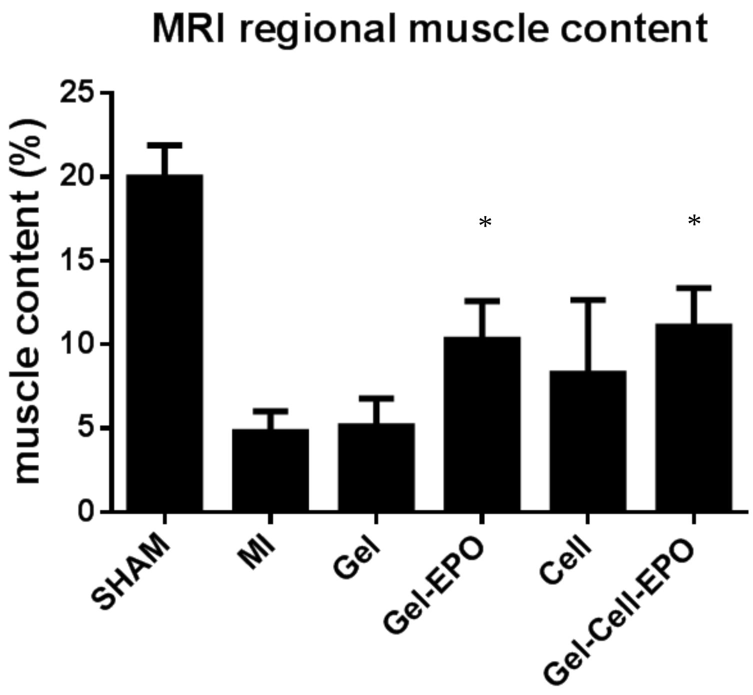

Figure S3. Regional muscle content within the infarct region

Myocardial viability within the apical (infarcted) region of the LV was measured as the non-enhanced area within apical LGE images and expressed as percentage viable tissue relative to the total left ventricular mass (to normalize for post infarct hypertrophy). All infarcted hearts had less viable tissue than sham animals. Hearts treated with GEL-EPO and GEL-CELL-EPO had significantly more viable tissue within the infarcted region than untreated infarcts. Data are presented as mean ± S.D. Statistically significant difference between group versus MI (* $p < 0.05$);

Figure S4

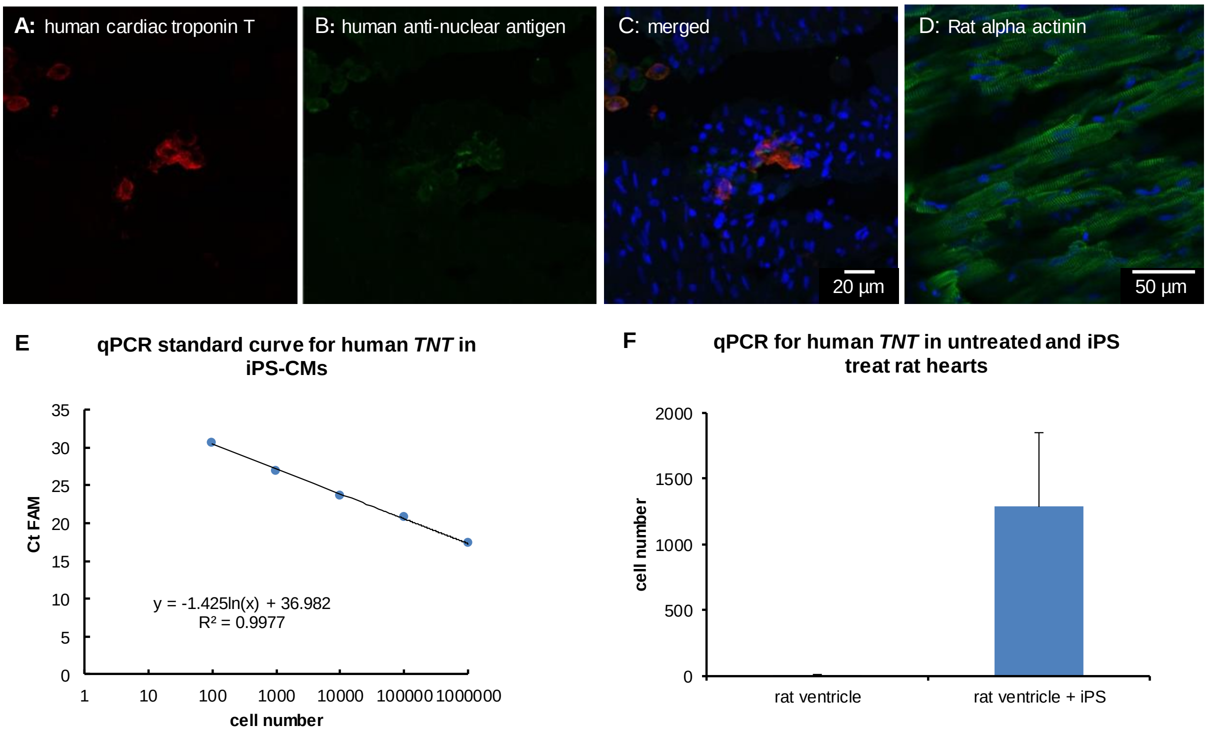

Figure S4. Immunohistochemical staining and qPCR of treated rat hearts

At 10 weeks after grafting immunohistochemical staining for human cardiac troponin T within iPS-CM grafted rat tissue was unable to identify any engrafted human cells. To confirm that human iPS-CMs could be detected within rat heart tissue using our protocol, we stained for human cardiac troponin T and human anti-nuclear antigen shortly after injection of iPS-CM into rat hearts. Human cardiac troponin T (A) and human anti-nuclear antigen (B) positive cells were readily identified within the tissue. Rat specific cardiomyocyte staining with alpha actinin is also presented (D). At 10 weeks after grafting qPCR for human cardiac markers within iPS-CM grafted rat tissue was unable to identify any engrafted human cells. To confirm that human iPS-CMs could be detected within rat heart tissue using our protocol, we generated a qPCR standard curve for human *TNT* from serial dilution on human iPS-CMs (E) and then used the same qPCR protocol to quantify cell content shortly after injection of 500 000 iPS-CM into rat hearts. Human *TNT* was detected in all hearts injected with iPS-CM (F), while untreated rat ventricular tissue had no human *TNT*. Data are presented as mean ± S.D.

Supplementary methods

Hydrogel preparation and materials

Four-arm polyethylene glycol (PEG) acrylate (PEG4A: Average MW = 23,300) and PEG dithiol (PEGDT: Average MW = 1,019) were purchased from Laysan Bio (Arab, AL, USA) and Sigma-Aldrich (Dorset, England) respectively. Basic PEG hydrogels were formed using a Michael addition reaction by adding PEGDT (dissolved in 1% triethanolamine) to PEG4A (dissolved in phosphate buffer saline).

Viscoelastic properties of the hydrogels were assessed using small amplitude oscillatory shear experiments on a rheometer (AR 2000ex, TA Instruments, Crawley, West Sussex, England) with parallel plate geometry (8 mm to 25 mm) at 37°C. Gel properties were examined by frequency sweep experiments at 1-10 hz (ω = 6.3–62.83 rad/s) at a fixed strain amplitude of 0.001 (0.1%) in order to ensure uniform mechanical properties were maintained at frequencies present in the human (1 hz, 6.3 rad/s) and rat (8 hz, 2.3 rad/s) heart.

Cell culture

Induced-pluripotent stem cell derived cardiomyocytes (iPS-CM) were purchased from Cellular Dynamics Inc (Madison, WI, USA) and have been extensively characterized by the supplier. From the 35 batches we received, the mean ± SD purity reported by suppliers was 98.2 ± 1.1% based on α-MHC staining, with no batches under 95%. Cells were thawed according to manufacturer's instructions and plated onto 0.1% (w/v) gelatin coated surfaces at a density of ~55,000 cells per cm^2. This allowed cultures to form a spontaneously beating syncytium of cardiomyocytes. Approximately 500,000 cells per rat were delivered by direct intramyocardial injections (see below). Cell viability after passing cells through the 27G needle used for injection was >90% as tested using trypan blue exclusion. Although this cell number is lower than that used in some other iPS-CM grafting studies, it is equivalent to many small animal and large animal experiments when scale is taken into consideration [1-4].

Animal model of myocardial infarction

All animal experiments were conducted under Home Office Project License 70/6568 and in accordance with local the Imperial College Ethics Committee and the ARRIVE Guidelines on animal research. Seven-week old male nude rats (approximately 150-200 g) were purchased from Charles River Limited (Germany), and allowed to acclimatize for 7 days prior to experiments. Prior to commencing surgery prophylactic antibiotics (Enrofloxacin 5 mg/kg intramuscular injection) were administered and analgesia (Buprenorphine 0.1 mg/kg intramuscular injection) was given. Induction of anaesthesia was performed by inhalation of a 5% Isoflurane in oxygen, then intubated and

ventilated using a Harvard Small Animal Ventilator (Model 683, Harvard Apparatus, Massachusetts, USA) at a ventilation rate of 80-90 breaths per minute, a tidal volume of 1.5-2.0 ml, and 2 - 2.5% isoflurane in oxygen. The thoracic cavity and heart were exposed via a left thoracotomy incision in the 3rd or 4th left intercostal space. A 7/0 prolene suture (Ethicon Inc., NJ, USA) on a round bodied curved needle was used to ligate the proximal part of the left anterior descending artery (LAD), approximately 1-2 mm distal to the inferior border of the left atrium, along an axis parallel to the atrioventricular groove. Successful ligation of the LAD was demonstrated by blanching and cyanosis of the myocardium distal to the tied suture, indicating myocardial ischemia. Sham surgery was performed by passing a suture and needle through the myocardium without ligation.

Injection of therapeutics

A 10% (w/v) 4-Armed PEG acrylate solution was prepared by 1:1 mixing of 20% 4-Armed PEG acrylate solution with cell suspension and the appropriate volume of 10% PEG dithiol was added to this mixture just prior to injection to initiate the gelling process. The final mixture was drawn up into a 1 ml syringe with 27G needle. Following successful infarction, hydrogels, cells, or a hydrogel and cell mixture was injected by 3 x 50 µl injections around the infarct border zone through a 27G needle. Maintenance anesthesia was reduced to 1.0% Isoflurane, and the chest wall was closed using a 4/0 Ethibond suture.

The experimental groups were as follows:

Sham: Sham operated control group that received PBS injection

MI: Myocardial infarction group that received PBS injection

Gel: MI group that received 10% PEG hydrogel injection

Gel-EPO: MI group that received 10% PEG hydrogel containing 1unit/ml EPO

Cell: MI group that received 5x10^5 iPS-CMs in PBS

Gel-Cell-EPO: MI group that received 10% PEG hydrogel containing 1unit/ml EPO and 5x10^5 iPS-CMs

Cardiac MRI

Anaesthetized rats were put under ECG with respiration monitoring and placed within a 4.7 Tesla DirectDrive Varian MRI System (Palo Alto, CA, USA). Cardiac and respiratory-gated cine-MRI was performed in the true short-axis orientation and covered the whole left ventricle (LV) (1.5 mm slice thickness, TE/TR 1.6/5 ms; 17.5° pulse; field of view 51.2 × 51.2 mm; matrix size 128 × 128; voxel size 400 × 400 × 1500 µm; 25 to 35 frames per cardiac cycle, 3 signal averages). Infarct size was

assessed by late gadolinuium enhanced (LGE) MRI performed 25 minutes after intraperitoneal injection of 0.5 mg/kg Gd-DTPA-BMA (Omniscan) using a multi slice inversion recovery sequence (1 mm slice thickness, TE/TR 1.6/3.2 ms; 90° excitation pulse; field of view 51.2 × 51.2 mm; matrix size 192 × 192; voxel size 266 × 266 × 1000 µm, 1 signal average) [5]. Data were analysed using Image J software (National Institutes of Health). End diastolic and end systolic volumes were measured by semi-automated segmentation of each slice and ejection fractions (EF) were calculated as (end diastolic volume - end systolic volume) / end diastolic volume. LV mass was measured by semi-automated segmentation of the myocardial area in all slices of the inversion recovery acquisition multiplied by the slice thickness (1 mm) and the specific gravity of the myocardium (1.05). Infarct mass was measured in every slice as the area of myocardial tissue with signal intensity >2 SD above signal intensity of remote tissue, multiplied by the slice thickness (1 mm) and the specific gravity of the myocardium (1.05). Infarct surface area was measured as the epicardial length of the enhanced tissue multiplied by the slice thickness, and was also expressed relative to the epicardial circumference of the whole LV (LGE surface area %). Myocardial viability within the apical (infarcted) region of the LV was measured as the non-enhanced area within apical LGE images and expressed as percentage viable tissue relative to the total left ventricular mass (to normalize for post infarct hypertrophy). MRI was performed within 48 hours of surgery and cell delivery, and repeated at 10 weeks. Primary outcome measures were LV end-diastolic and end-systolic chamber volumes, stroke volume and EF and LGE infarct size. MRI acquisition and analysis was performed by an investigator blinded to the experimental groups.

Histology

Ten weeks after infarction, animals were sacrificed; hearts were fixed by intraventricular infusion of 10% (v/v) formalin, explanted, and kept in 10% formalin for 24 hours followed by 30% (w/v) sucrose solution for at least 24 hours. Hearts were embedded in Optimal Cutting Temperature compound, frozen in liquid nitrogen-cooled isopentane baths and 10 µm sections were cut using a cryotome. For immunostaining studies, negative control samples were taken from the MI group, and positive control samples were from human tissue taken from patients with dilated cardiomyopathy, as well as iPS-CM grown on glass coverslips. Primary antibodies for human ANA (1 in 200, MAB1281, Millipore) and troponin (1in 250, AB45932, Abcam) were then incubated for 2 hours, washed in D-PBS and then incubated with secondary antibodies (Alexa fluor 488 and 546, 1 in 500) for 1 hour. After further washing, sections were incubated with Hoechst (1 in 1000) for 10 minutes.

Images of the infarct zone (x4 magnification) were analyzed using ImageJ software. This allowed measurement of infarct thickness, as well as measurement of the proportion of the infarct stained red by Massons Trichrome. This allowed calculation of the amount of muscle fibres present in the infarct zone. Fluorescence microscopy was used to image samples labeled with immunostaining.

Quantitative Polymerase Chain Reaction (qPCR)

The infarct zone and remaining left ventricular wall was excised from ~3 mm thick frozen heart sections and approximately 30 mg of each were placed separately into ice-cold TRI Reagent (Sigma-Aldrich). Tissue samples were disrupted and homogenized in the TRI Reagent solution using a rotor-stator homogenizer. Following homogenization, total RNA was extracted from cardiac tissue samples using a RNeasy Mini Kit (Qiagen) according to the manufacturer's specifications and cDNA was reverse transcribed from normalized amounts of RNA, as determined by absorbance at 260 nm (A_{260}) in a NanoDrop 8000 spectrophotometer. A similar protocol was used for the acute cell retention studies presented in Figure S4 except 50 – 100 mg of tissue was used. All RNA procedures were conducted in an RNase-free environment. All freshly synthesized cDNA was stored overnight at 4°C. The following day, real-time PCR was performed in technical triplicate using pre-validated TaqMan® assays from Life Technologies™ in a Rotor Gene 6000 (Corbett Life Science) according to the manufacturers' instructions.

Infarct zones and remaining left ventricular areas were analyzed separately. Negative control samples were taken from the sham and MI groups. Positive control samples used were human samples from patients with dilated cardiomyopathy. qPCR was performed for an assortment of early and late cardiac markers, including atrial natriuretic factor (*NPPA,* Hs00383230_g1*)*, myosin-6 (*MYH6,* HS01101425_M1), myosin-7 (*MYH7,* HS01110632_M1), alpha-1A and 1B adrenergic receptors (*ADRA1a,* HS00169124_M1 and *ADRA1b,* HS00171263_M1) and cardiac troponin T (*TNT*, HS00165960_M1).

Statistical Analysis

Results were tabulated using Microsoft Excel and Graphpad Prism. A one-way between groups ANOVA ($\alpha = 0.05$) was performed for group comparison. If the results of the ANOVA were found to be significant, post-hoc analysis was performed using the Tukey multiple comparisons test to compare results between all groups. Data are presented as mean ± SEM.

References

1. Miao Q, Shim W, Tee N, Lim SY, Chung YY, Ja KP, Ooi TH, Tan G, Kong G, Wei H *et al*: **iPSC-derived human mesenchymal stem cells improve myocardial strain of infarcted myocardium**. *Journal of cellular and molecular medicine* 2014, **18**(8):1644-1654.
2. Citro L, Naidu S, Hassan F, Kuppusamy ML, Kuppusamy P, Angelos MG, Khan M: **Comparison of human induced pluripotent stem-cell derived cardiomyocytes with human mesenchymal stem cells following acute myocardial infarction**. *PloS one* 2014, **9**(12):e116281.
3. Shiba Y, Gomibuchi T, Seto T, Wada Y, Ichimura H, Tanaka Y, Ogasawara T, Okada K, Shiba N, Sakamoto K *et al*: **Allogeneic transplantation of iPS cell-derived cardiomyocytes regenerates primate hearts**. *Nature* 2016, **538**(7625):388-391.
4. Li X, Zhang F, Song G, Gu W, Chen M, Yang B, Li D, Wang D, Cao K: **Intramyocardial Injection of Pig Pluripotent Stem Cells Improves Left Ventricular Function and Perfusion: A Study in a Porcine Model of Acute Myocardial Infarction**. *PloS one* 2013, **8**(6):e66688.
5. Stuckey DJ, McSweeney SJ, Thin MZ, Habib J, Price AN, Fiedler LR, Gsell W, Prasad SK, Schneider MD: **T(1) mapping detects pharmacological retardation of diffuse cardiac fibrosis in mouse pressure-overload hypertrophy**. *Circ Cardiovasc Imaging* 2014, **7**(2):240-249.
